# Effective Thermal Conductivity of Hybrid AlN/Graphene Polymer Matrix Composites: A Hierarchical Micromechanical Model

**DOI:** 10.3390/polym18212716

**Published:** 2026-11-02

**Authors:** Usama Umer, Mustufa Haider Abidi, Syed Hammad Mian, Khaja Moiduddin, Abdulrahman Bin Jumah

**Affiliations:** 1 Advanced Manufacturing Institute, King Saud University, Riyadh 11421, Saudi Arabia; 2 Chemical Engineering Department, College of Engineering, King Saud University, Riyadh 11421, Saudi Arabia

**Keywords:** thermal conductivity, hybrid polymer composite, aluminum nitride, graphene nanoplatelets, micromechanical modeling

## Abstract

The demand for lightweight polymer composites with enhanced thermal conductivity is increasing in advanced thermal-management applications. This study develops a hierarchical micromechanical model to predict the effective thermal conductivity of hybrid polymer composites reinforced with aluminum nitride (AlN) particles and graphene nanoplatelets (GNPs). The modeling procedure is performed in two successive stages. First, the effective thermal conductivity of the GNP/polymer matrix is determined by considering the graphene volume fraction, length, thickness, and flatness ratio as well as the interfacial thermal resistance. The resulting graphene/polymer phase is then treated as an effective matrix for AlN micro-reinforcement. An analytical micromechanics formulation on the basis of the representative volume element (RVE) is used to determine the thermal conductivity of the final hybrid composite. The results show that adding graphene considerably enhances the thermal conductivity of the AlN/polymer composite. Larger graphene length also improves the heat-transfer characteristics of the hybrid polymer composite. In contrast, increasing graphene thickness reduces the thermal conductivity enhancement. It is found that the AlN content and aspect ratio strongly affect the AlN/GNP/polymer composite thermal conductivity. The proposed model provides useful guidance for designing thermally conductive polymer composite structures.

## 1. Introduction

The continuous advancement of modern technologies, particularly in electronics, aerospace, automotive, and energy systems, has intensified the need for lightweight materials with efficient thermal-management capabilities. In high-power-electronic and engineering systems, effective heat dissipation is essential for controlling operating temperatures, maintaining functional performance, and extending service life [1,2,3]. Polymer materials are attractive for such applications due to their low density, simplified processing, mechanical flexibility, corrosion resistance, and design versatility. However, their inherently low thermal conductivity remains a major limitation for applications requiring rapid and efficient heat transfer [4,5,6,7]. Therefore, improving the thermal conductivity of polymer-based materials has become an important research objective in the development of advanced thermal-management materials.

A common method for enhancing the thermal conductivity of polymers is the incorporation of thermally conductive micro- and nanofillers. Various reinforcements, including short carbon fibers (SCFs), graphene nanoplatelets (GNPs), carbon nanotubes (CNTs), and other carbon-based nanomaterials, have been extensively investigated for this purpose [8,9,10,11,12,13,14,15,16,17,18,19,20,21,22,23]. These fillers can facilitate heat transport through the polymer matrix by providing preferential pathways for thermal energy propagation. Notably, graphene-based nanofillers have garnered significant research interest owing to their exceptionally high intrinsic thermal conductivity, theoretically reported to be approximately 3000–5000 W/m·K [24]. However, the thermal conductivity of graphene/polymer composites is not determined solely by the intrinsic conductivity of graphene. Instead, it is strongly influenced by factors such as filler concentration, geometrical characteristics, filler–matrix interfacial resistance, and the creation of continuous heat-conduction pathways.

The effectiveness of conductive fillers has been demonstrated experimentally in a number of polymer composite systems. Wang et al. [25], for example, reported that incorporating GNPs into an epoxy matrix resulted in a thermal-conductivity enhancement of up to 627% at an 8 wt% loading compared with neat epoxy. This improvement was attributed to the high thermal conductivity of graphene and the establishment of effective heat-transfer pathways within the matrix. Zhang et al. [26] reported that the addition of only 2 wt% CNTs increased the thermal conductivity of the resulting composite from 0.15 to 0.8 W/m·K. Such improvements were primarily associated with the formation of interconnected conductive networks and enhanced heat transport through the filler phase.

Despite the considerable enhancement achievable using individual fillers, the use of a single reinforcement may not fully exploit the potential for optimizing heat transfer in polymer composites. This limitation has motivated increasing interest in hybrid composites containing two or more fillers with different geometries, dimensions, and thermal characteristics [27,28,29,30,31,32,33]. The combination of different reinforcements can provide complementary heat-transfer pathways and, under appropriate conditions, produce a synergistic thermal response. For instance, Li et al. [34] investigated epoxy composites containing hybrid GNP/CNT fillers and reported a substantial improvement in thermal conductivity, which was primarily attributed to the development of interconnected heat-conduction networks. Pradhan et al. [35] similarly demonstrated that hybrid graphite-flake/CNT reinforcement in polycarbonate could simultaneously improve thermal conductivity, mechanical strength, electrical conductivity, and electromagnetic-interference shielding. These studies highlight the potential of hybridization as an effective strategy for overcoming the limitations associated with individual fillers. In addition to carbon-based reinforcements, thermally conductive ceramic fillers have attracted significant interest because they can provide enhanced heat-transfer capability. Among these materials, aluminum nitride (AlN) is particularly promising owing to its high thermal conductivity, relatively low density, and good thermal stability [36]. A literature survey showed that no systematic investigation has been performed on the thermal conductivity of AlN/graphene-reinforced polymer matrix composites considering important microstructures. The main novelty of this research lies in the development of a multistep homogenization procedure that combines appropriate micromechanical models to account for the contributions of nanoscale GNPs and microscale AlN particles within a unified framework.

Therefore, a hierarchical micromechanical framework is developed to evaluate the effective thermal conductivity of a hybrid polymer composite reinforced with GNPs and AlN microscale particles in this study. The proposed approach considers two reinforcement phases through a sequential homogenization procedure. First, the effective thermal conductivity of the GNP/polymer composite is determined by accounting for the thermal characteristics and geometrical parameters of the GNPs, as well as the thermal interaction between the graphene fillers and the polymer matrix. The resulting GNP/polymer composite is subsequently regarded as an effective matrix for the incorporation of AlN particles. In the second stage, the effective thermal conductivity of the resulting GNP/AlN/polymer hybrid composite is evaluated by considering the contribution of the AlN reinforcement. The proposed hierarchical framework provides new insights into the thermal transport behavior of GNP/AlN/polymer hybrid composites and establishes a predictive basis for optimizing filler content, geometry, and interfacial characteristics, thereby facilitating the rational design of high-performance thermally conductive polymer composites.

## 2. Micromechanical Theory

### 2.1. GNP-Filled Polymer Materials

In this section, we formulate an analytical model within the micromechanical framework for predicting the thermal conductivity of GNP-reinforced polymer composites. The composite system is assumed isotropic with GNP inclusions randomly oriented throughout the matrix.

First, a two-phase composite consisting of a homogeneous matrix containing randomly distributed filler particles is considered. Each particle is assumed to occupy a finite region within the matrix, where the local thermal conductivity may vary spatially from one point to another and particle–particle interactions are neglected. The thermal conductivity variation in the composite is formulated as $K^{*}=K^{0}+K^{\prime }(r)$, where $K^{0}$ is a constant section of a homogeneous medium and $K^{\prime }(r)$ specifies an arbitrary fluctuating section.

Based on Green’s function with G corresponding to the homogeneous reference medium, together with the transition matrix T characterizing the heterogeneous composite, the spatial distribution of the temperature gradient can be rigorously determined. Consequently, the effective thermal conductivity $K^{*}$ of the composite is obtained in the following form [19,37]:

(1) $$K^{*}=K_{m}+\langle T\rangle {\left(I+\langle GT\rangle \right)}^{-1}$$

Here, *I* denotes the unit tensor, while ⟨ ⟩ represents the spatial averaging operator. Under the assumption that multiple scattering interactions between the particles can be neglected, the transition matrix T can be approximated in the following form:

(2) $$T=\sum_{n}K_{n}^{\prime }{\left(1-GK_{n}^{\prime }\right)}^{-1}$$

By selecting the matrix as a homogeneous medium, such that $K^{0}=K_{m}$, where $K_{m}$ denotes the matrix thermal conductivity, the equivalent thermal conductivity $K^{*}$ of the composite containing ellipsoidal inclusions can be written in the following form:

(3) $$\frac{K^{*}}{K_{m}}=\frac{\left[2A_{x}\left(L_{x}-1\right)+A_{z}\left(L_{z}-1\right)\right]/f/3-1}{\left(2A_{x}L_{x}+A_{z}L_{z}\right)f/3-1}$$

in which

(4) $$\begin{array}{c} A_{x}=\frac{K_{x}-K_{m}}{K_{m}+L_{x}\left(K_{x}-K_{m}\right)}\\ A_{z}=\frac{K_{z}-K_{m}}{K_{z}+L_{z}\left(K_{z}-K_{m}\right)} \end{array}$$

**Figure 1:**
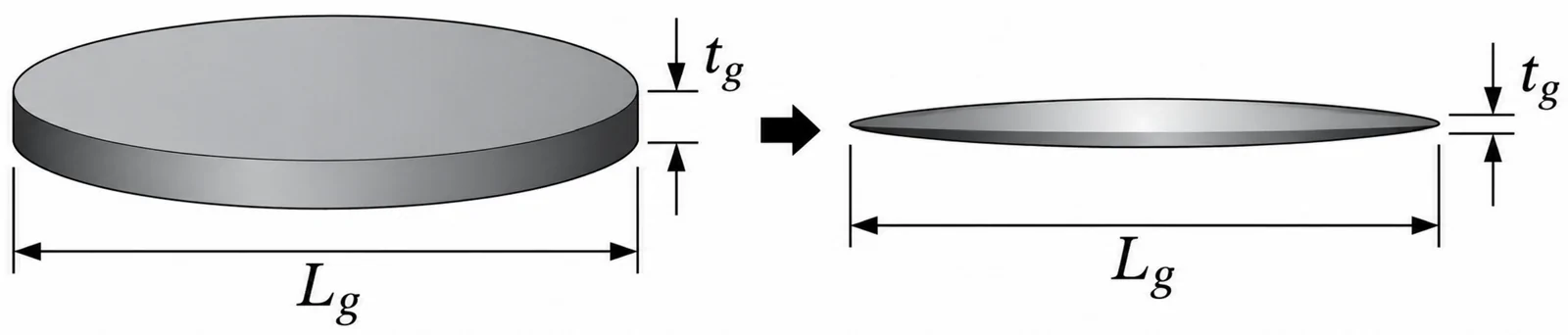
An illustration of the GNP filler as an oblate spheroid during the modeling.

Here, $K_{x}$ and $K_{z}$ correspond to the thermal conductivity components of the ellipsoidal particles in the transverse and longitudinal directions, respectively. The parameter f represents the volume fraction of the filler phase. It is noted that GNP is incorporated as an equivalent oblate spheroid possessing a large aspect ratio $P=L_{g}/t_{g}$ ($L_{g}$ is the length and $t_{g}$ is the thickness), as depicted in Figure 1. In the above equations, $L_{x}$ and $L_{z}$ represent the geometrical factors of the ellipsoidal fillers, which are governed by the particle aspect ratio P and can be determined from the following expressions:

(5) $$\begin{array}{c} L_{x}=\frac{P^{2}}{2(P^{2}-1)}-\frac{P}{2{\left(P^{2}-1\right)}^{3/2}}\cosh^{-1}P,\;P>1\\ L_{z}=1-2L_{x} \end{array}$$

In general, the pronounced thermal anisotropy of GNPs enables them to behave as highly efficient two-dimensional thermal conductors, with negligible heat-flux losses during long-range heat transport. For a non-flat GNP, such as a wrinkled or folded nanoplatelet, subjected to a temperature difference of $∆T=T_{2}-T_{1}$ between its two ends, the heat flux Q through the GNP can be expressed as follows [19]:

(6) $$Q=\frac{K_{g}∆T}{L_{g}}$$

For the purpose of thermal analysis, the GNP is represented by an equivalent flat disk with a thermal conductivity of $K_{g}^{e}$. The equivalent disk is defined to maintain the same heat flux Q through the two ends of the graphene over the characteristic distance $L_{g}^{e}$. Accordingly, the heat flux Q across the equivalent planar graphene can be determined from the following expression:

(7) $$Q=\frac{K_{g}^{e}∆T}{L_{g}^{e}}$$

Because the two relations, Equations (6) and (7) are equivalent, one can write the following:

(8) $$\frac{K_{g}^{e}}{L_{g}^{e}}=\frac{K_{g}}{L_{g}}$$

Consequently, the equivalent length $\left(L_{g}^{e}\right)$ and the thermal conductivity $\left(K_{g}^{e}\right)$ of the flat GNP are determined from the following expressions, respectively:

(9) $$\begin{array}{c} L_{g}^{e}=\eta L_{g}\\ K_{g}^{e}=\eta K_{g} \end{array}$$

In this expression, η represents the average flatness ratio of the GNPs and is defined mathematically by the following:

(10) $$\eta =\int_{0}^{\infty }P\left(L_{g}\right)\;\eta \left(L_{g}\right)\;L_{g}\;dL_{g}$$

where $P(L_{g})$ denotes the length distribution function of GNPs. The intrinsic thermal conductivity of graphene, $K_{g}$, exhibits a pronounced dependence on the number of graphene layers, n, which is directly related to the nanoplatelet thickness, $t_{g}$, as reported in previous studies [19,38]. The following expression is used for the thickness-dependent thermal conductivity, $K_{g}$, as follows [19,38]:

(11) $$K_{g}=4058\times {\left(\frac{t_{g}}{3.4\times 10^{-10}}\right)}^{-1/2}$$

Accordingly, the intrinsic thermal conductivity $K_{g}$ can be estimated as a function of the GNP thickness ($t_{g}$). Furthermore, the thermal resistance at the filler–matrix interface, $\left(R_{K}\right)$, plays a critical role in determining the heat-transfer characteristics of nanofiller-reinforced composites. To incorporate the influence of interfacial thermal resistance $R_{K}$, the GNP is modeled as a solid filler coated by a very thin interfacial thermal barrier layer with thickness δ and conductivity $K_{S}$ as a composite unit cell [19,39]. $R_{K}$ can be expressed as $R_{K}=\lim_{\delta \to 0,K_{S}\to 0}\delta /K_{S}$.

Accordingly, the interfacial thermal resistance effect is incorporated into the formulation, yielding the following relation for the GNP thermal conductivity, ($K_{g}^{eff}$):

(12) $$K_{g}^{eff}=\frac{K_{g}}{2R_{K}K_{g}/L_{g}+1}$$

By incorporating the thickness dependence of $K_{g}$, the interfacial thermal resistance $\left(R_{K}\right)$ and in particular, the flatness ratio of the GNPs (η), the effective thermal conductivity of the GNP-reinforced composite can be obtained from the following expression:

(13) $$\frac{K^{*}}{K_{m}}=\frac{3+2\eta^{2}f/\left[K_{m}\left(2R_{K}/L_{g}+13.4\sqrt{t_{g}}\right)\right]}{3-\eta f}$$

The general formulation presented in Equation (13) incorporates the contributions of GNP thickness, length, interfacial thermal resistance, and flatness ratio to the thermal response.

### 2.2. Thermal Conductivity of AlN-Reinforced Composites

In the second phase of the modeling, AlN microscale particles are introduced into the GNP/polymer matrix to form a ternary composite. A two-phase composite medium is considered, comprising a homogeneous matrix and a finite number of inclusions embedded within it. The heat-transfer response of this heterogeneous system is governed by the heat-conduction equation, which accounts for the spatial variation in thermal properties and can be written in the following general form [40]:

(14) $$\left(\rho_{i}c_{i}\right)\frac{\partial T_{i}}{\partial t}=\nabla \left(k_{i}\nabla T_{i}\right)\;\;in\;\;\Omega_{i}\left(i=1,2\right)$$

where $\rho_{i}$, $c_{i}$, $T_{i}$, t and $k_{i}$ stand for the density, specific heat, temperature, time and the thermal conductivity, respectively, and the subscript *i* denotes component *i* within the composite, and $\Omega_{i}$ specifies the domain of component i (*i* = 1 for the reinforcement and i = 2 for the matrix). To characterize heat transfer across the interface between the two constituent phases, the temperature and heat-flux conditions must be appropriately defined. Accordingly, for a two-phase composite material, the interfacial relations are written as follows:

(15) $$\left(k_{i}\right)\nabla T_{i}=h\left(T_{2}-T_{1}\right)\;in\;\Gamma_{12}\left(i=1,2\right)$$

In this formulation, h is the convective coefficient, and $\Gamma_{12}$ represents the interface between component 1 and component 2 [40].

**Figure 2:**
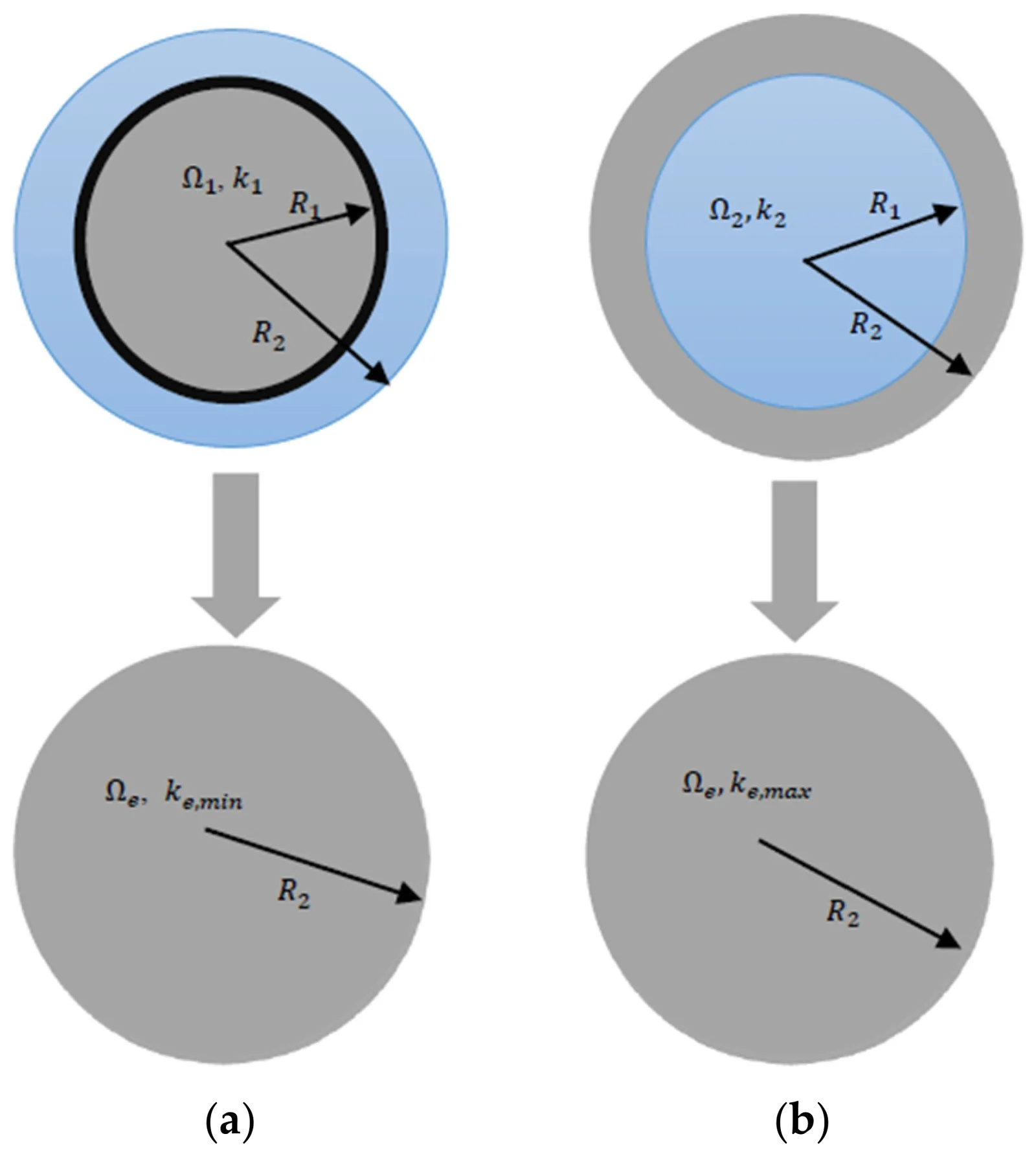
Schematic representation of the spherical model for two-phase composite systems: (**a**) inclusions dispersed within a continuous matrix phase, and (**b**) a continuous inclusion phase surrounding discrete regions of the matrix.

Figure 2a illustrates the representative volume element (RVE) employed in the spherical model for the two-phase composite. The RVE consists of a spherical reinforcement phase enclosed by an outer shell representing the surrounding matrix medium. Accordingly, the respective volume fractions of the reinforcement ($V_{1}$) and matrix ($V_{2}$) phases within the RVE are given, respectively, by the following:

$$V_{1}={\left(\frac{R_{1}}{R_{2}}\right)}^{3}$$

(16) $$V_{2}=1-{\left(\frac{R_{1}}{R_{2}}\right)}^{3}$$

The parameters $R_{1}$ and $R_{2}$ denote the radius of inner reinforcement and outer radius of the representative spherical shell, respectively. It is supposed that the interface between the inclusion and medium does not absorb thermal energy. Consequently, the density and specific heat capacity are obtained, respectively, as follows [40]:

(17) $$\rho_{e}=\rho_{1}V_{1}+\rho_{2}V_{2}$$

(18) $$c_{e}=\frac{c_{1}\rho_{1}V_{1}+c_{2}\rho_{2}V_{2}}{\rho_{1}V_{1}+\rho_{2}V_{2}}$$

where $\rho_{1}$ and $c_{1}$ denote the density and specific heat of the inclusion, and $\rho_{2}$ and $c_{2}$ denote the density and specific heat of the medium, in sequence.

The effective thermal conductivity of a two-phase composite material is obtained by replacing the heterogeneous system with an equivalent homogeneous medium that reproduces its overall temperature distribution and heat-transfer behavior. Within this framework, the composite is idealized as a concentric spherical configuration, as illustrated in Figure 2a, consisting of an inclusion embedded in a surrounding matrix. Assuming steady-state heat conduction, the temperature field in each phase is governed by the following heat-conduction equation [40]:

(19) $$\frac{1}{r^{2}}\frac{\partial }{\partial r}\left(k_{i}r^{2}\frac{\partial T_{i}}{\partial r}\right)+\frac{1}{r^{2}\sin\theta }\frac{\partial }{\partial \theta }\left(k_{i}\sin\theta \frac{\partial T_{i}}{\partial \theta }\right)=0\;in\;\Omega_{i}\left(i=1,2\right)$$

in which r,θ and ϕ denote the spherical coordinates, assuming the coordinate origin enforced at the common center of the spheres, $\Omega_{1}$, specifies the domain of $r<R_{1}$, and $\Omega_{2}$ denotes the domain of $R_{1}<r<R_{2}$. The $x_{3}$-axis is adopted as the axis of axial symmetry, with $x_{3}=rcos\theta$. The boundary condition of temperature or heat flux can be expressed at its infinity $\left(x_{3}\to \infty \right)$. The overall solution to Equation (19) is written in the following form:

(20) $$T_{i}\left(r,\theta ,\phi \right)=\left(A_{i}r+\frac{B_{i}}{r^{2}}\right)\cos\theta \;in\;\Omega_{i}\left(i=1,2\right)$$

in which $A_{i}$ and $B_{i}$ (i=1,2) denote the constants that should be calculated. To ensure a finite temperature at the center of the spherical inclusion, $B_{1}=0$. Constant $A_{2}$ is calculated on the basis of the enforced remote boundary condition. Also, $A_{1}$ and $B_{2}$ are obtained by enforcing the thermal condition at the interface ($r=R_{1}$), following Equation (15), which is written as follows:

(21) $${\left[k_{1}\frac{\partial T_{1}}{\partial r}\right]}_{r=R_{1}}={\left[k_{2}\frac{\partial T_{2}}{\partial r}\right]}_{r=R_{1}}$$

(22) $${\left[k_{1}\frac{\partial T_{1}}{\partial r}\right]}_{r=R_{1}}={\left[h\left(T_{2}-T_{1}\right)\right]}_{r=R_{1}}$$

By inserting Equation (20) into Equations (21) and (22), the resulting expressions for $A_{1}$ and $B_{2}$ are obtained as follows:

(23) $$A_{1}=\frac{3A_{2}}{\left(2+\frac{2k_{1}}{hR_{1}}+\frac{k_{1}}{k_{2}}\right)}$$

(24) $$\frac{B_{2}}{R_{1}^{3}}=\frac{\left(1+\frac{k_{1}}{hR_{1}}-\frac{k_{1}}{k_{2}}\right)A_{2}}{\left(2+\frac{2k_{1}}{hR_{1}}+\frac{k_{1}}{k_{2}}\right)}$$

Next, an RVE consisting of a single homogeneous material with the same dimensions as those depicted in Figure 2a is considered. The solution of Equation (19) within this RVE $\left(\Omega =\Omega_{1}+\Omega_{2}\right)$ is then written as follows:

(25) $$T_{e}\left(r,\theta ,\phi \right)=\left(A_{e}r+\frac{B_{e}}{r^{2}}\right)\cos\theta \;in\;\Omega$$

where $T_{e}$ denotes the temperature, and $A_{e}$ and $B_{e}$ are the constants to be calculated. The equivalence between the homogeneous single-phase model and the original two-phase composite is established by requiring identical thermal conditions at the external surface of the sphere. Specifically, continuity of both the temperature field and radial heat flux at $r=R_{2}$ leads to the following two governing relations:

(26) $${\left[T_{e}\right]}_{r=R_{2}}={\left[T_{2}\right]}_{r=R_{2}}$$

(27) $${\left[k_{e}\frac{\partial T_{e}}{\partial r}\right]}_{r=R_{2}}={\left[k_{2}\frac{\partial T_{2}}{\partial r}\right]}_{r=R_{2}}$$

Here, $k_{e}$ denotes the effective thermal conductivity. Upon inserting Equations (20) and (25) into Equations (26) and (27), the singularity condition at the origin requires $B_{e}=0$. Accordingly, the resulting relations take the following form [40]:

(28) $$k_{e,min}=k_{e}=k_{2}\frac{\left(2+\frac{2k_{1}}{hR_{1}}+\frac{k_{1}}{k_{2}}\right)-2V_{1}\left(1+\frac{k_{1}}{hR_{1}}-\frac{k_{1}}{k_{2}}\right)}{\left(2+\frac{2k_{1}}{hR_{1}}+\frac{k_{1}}{k_{2}}\right)+V_{1}\left(1+\frac{k_{1}}{hR_{1}}-\frac{k_{1}}{k_{2}}\right)}$$

Equation (28) provides the thermal conductivity of the two-phase composite. In this equation, $k_{e}$ corresponds to the lower bound of the effective thermal conductivity and is therefore denoted by $k_{e,min}$. To determine the upper bound, the inclusions are assumed to form a fully percolated network. Under this assumption, the spatial arrangement of the two phases in the spherical method depicted in Figure 2a is reversed: the inner sphere represents the matrix phase, while the surrounding shell corresponds to the inclusion phase, as illustrated in Figure 2b. Under such a configuration, the upper bound of the thermal conductivity of the two-phase composite is determined directly from Equation (28) by swapping $k_{1}↔k_{2}$ and $V_{1}↔(1-V_{1})$ as follows [40]:

(29) $$k_{e,max}=k_{e}=k_{1}\frac{\left(2+\frac{2k_{2}}{hR_{1}}+\frac{k_{2}}{k_{1}}\right)-2\left({1-V}_{1}\right)\left(1+\frac{k_{2}}{hR_{1}}-\frac{k_{2}}{k_{1}}\right)}{\left(2+\frac{2k_{2}}{hR_{1}}+\frac{k_{2}}{k_{1}}\right)+\left({1-V}_{1}\right)\left(1+\frac{k_{2}}{hR_{1}}-\frac{k_{2}}{k_{1}}\right)}$$

**Figure 3:**
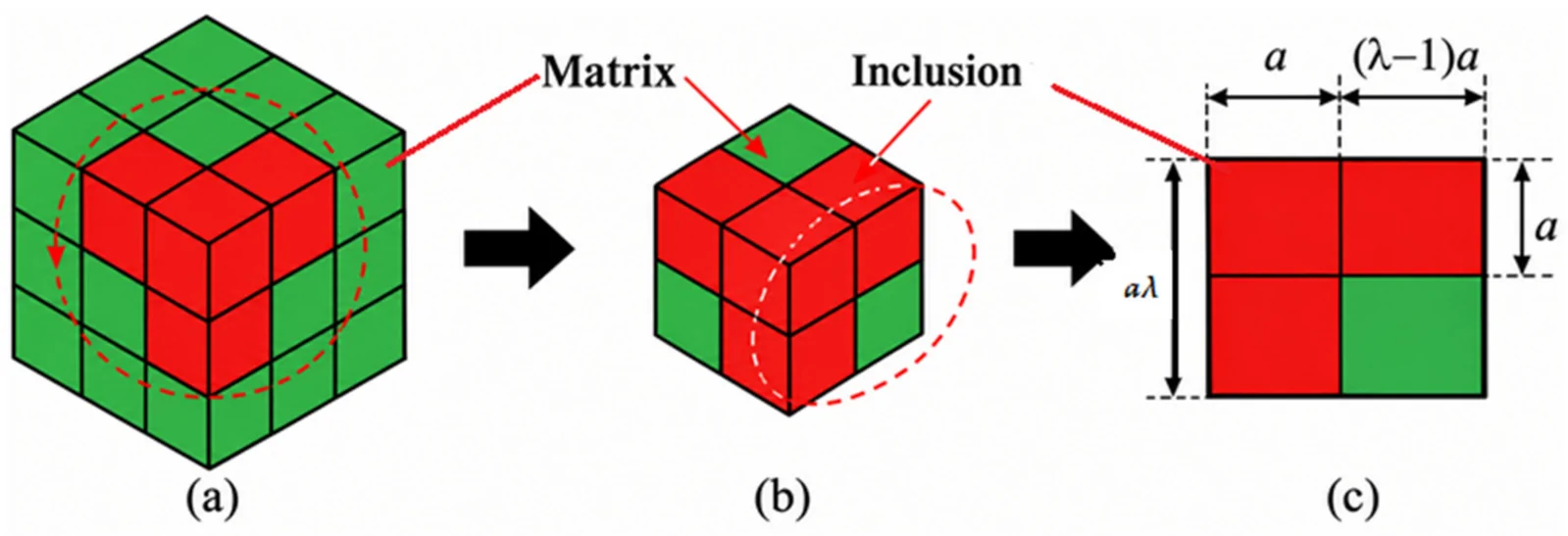
An illustration of the filled composite in which the matrix and inclusion are specified by green and red, respectively. (**a**) A unit volume cube of the composite RVE; (**b**) a sub-domain containing an inclusion phase; and (**c**) its side view.

The random dispersion of inclusions is represented by considering a unit-volume RVE, as shown in Figure 3. In this figure, λ and a denote the aspect ratio of reinforcement and virtual length parameter, respectively. To simplify the analysis, the inclusions are idealized as solid fillers, while their cross-sectional effects are neglected. For a specified λ, the corresponding virtual length a is determined from the inclusion volume fraction within the composite using Equation (30) [40]. Based on the configuration illustrated in Figure 3a, the corresponding relations can be determined as follows:

(30) $$\begin{array}{c} \mathrm{If}\;\lambda a<1\\ V_{1}=3\lambda a^{3}-2a^{3}=\left(3\lambda -2\right)a^{3}\\ V_{m1}={\left(\lambda a\right)}^{3}-\left(3\lambda -2\right)a^{3}=\left(\lambda^{3}-3\lambda +2\right)a^{3}\\ V_{m2}=1-{\left(\lambda a\right)}^{3}\\ \mathrm{If}\;\lambda a=1\\ V_{1}=3\lambda a^{3}-2a^{3}=\left(3-2a\right)a^{2}\\ V_{m1}=1-\left(3-2a\right)a^{2}\\ V_{m2}=0 \end{array}$$

Here $V_{m1}$ denotes the volume of the medium (or matrix) in the sub-domain indicated in Figure 3b; $V_{m2}$ represents the volume of the medium in the unit volume of the cube subtract the sub-domain. Also, $V_{m1}+V_{m2}=V_{2}$ specifies the volume fraction of the medium in the composite material. Following the approach presented in Ref. [40], the thermal conductivity of the RVE is determined through a two-stage procedure. In the first stage, the thermal conductivity of the sub-domain depicted in Figure 3b is determined using Equation (29) where the matrix region ($V_{1m}$) is surrounded by the inclusion phase ($V_{1}$) as follows:

(31) $$k_{e}^{1}=k_{AIN}\frac{\left(2+\frac{2k_{m}}{hR_{1}}+\frac{k_{m}}{k_{AlN}}\right)-2\left(1-\frac{V_{1}}{V_{1}+V_{m1}}\right)\left(1+\frac{k_{m}}{hR_{1}}-\frac{k_{m}}{k_{AlN}}\right)}{\left(2+\frac{2k_{m}}{hR_{1}}+\frac{k_{m}}{k_{AlN}}\right)+\left(1-\frac{V_{1}}{V_{1}+V_{m1}}\right)\left(1+\frac{k_{m}}{hR_{1}}-\frac{k_{m}}{k_{AlN}}\right)}$$

Here $k_{e}^{1}$ denotes the thermal conductivity of the sub-domain and $k_{AlN}$ and $k_{m}$ denote the thermal conductivities of the AlN microscale particles and the surrounding matrix, respectively. In the second stage, the effective thermal conductivity of the RVE illustrated in Figure 3a is determined using Equation (28) where the previously homogenized sub-domain, with a volume fraction of $\left(V_{1}+V_{1m}\right)$ and the thermal conductivity, $k_{e}^{1}$, is embedded within the surrounding matrix phase ($V_{2m}$) as follows [40]:

(32) $$K_{C}=k_{m}\frac{\left(2+\frac{2k_{e}^{1}}{hR_{1}}+\frac{k_{e}^{1}}{k_{AIN}}\right)-2\left(V_{1}+V_{m1}\right)\left(1+\frac{k_{e}^{1}}{hR_{1}}-\frac{k_{e}^{1}}{k_{AIN}}\right)}{\left(2+\frac{2k_{e}^{1}}{hR_{1}}+\frac{k_{e}^{1}}{k_{AIN}}\right)+\left(V_{1}+V_{m1}\right)\left(1+\frac{k_{e}^{1}}{hR_{1}}-\frac{k_{e}^{1}}{k_{AIN}}\right)}$$

Here, $K_{C}$ is the effective thermal conductivity of the RVE.

## 3. Results and Discussion

### 3.1. Validation

**Figure 4:**
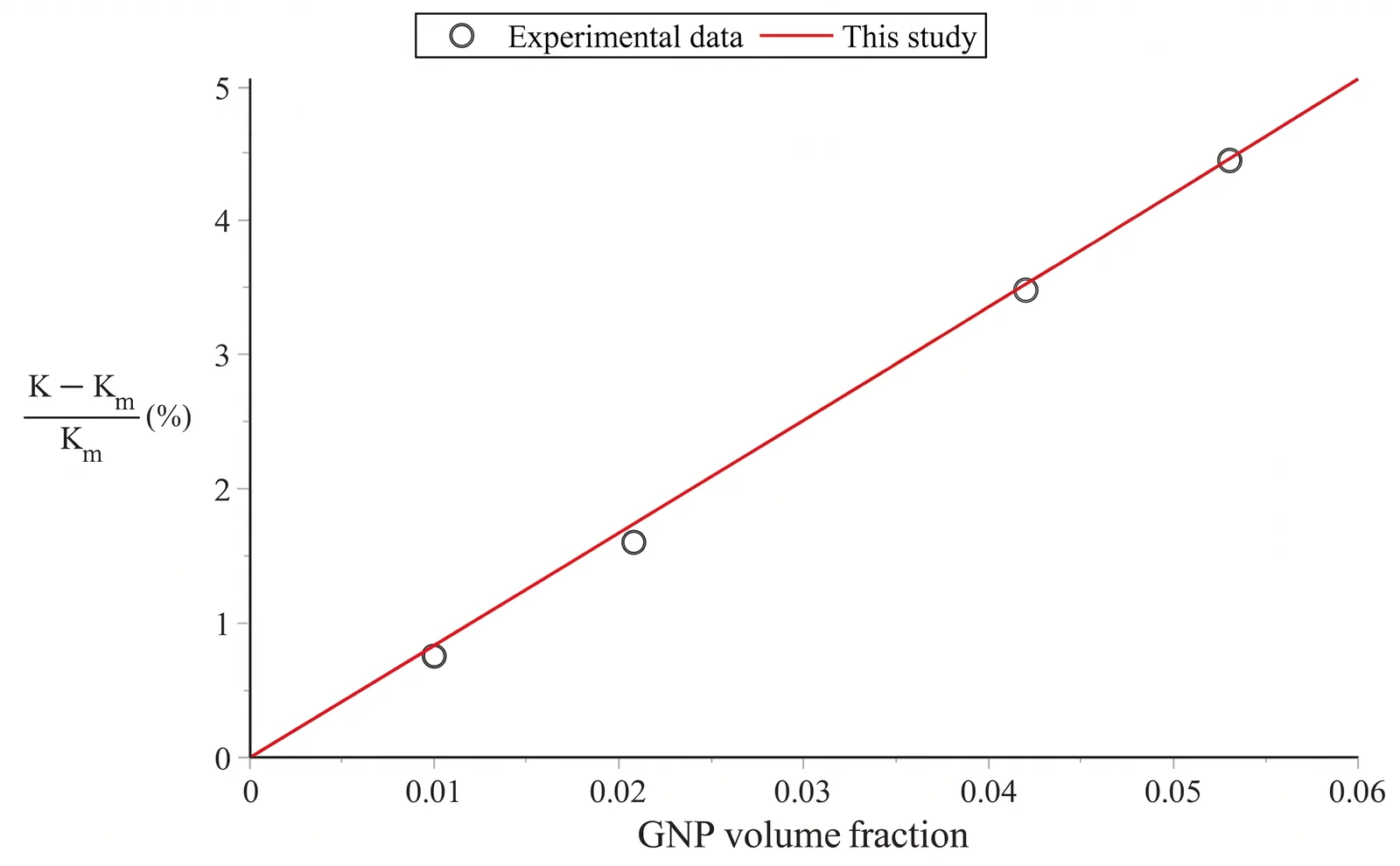
Validation of the proposed model through comparison with the experimental data for multilayer GNP-filled composites reported in Ref. [41].

The accuracy, predictive capability, and robustness of the proposed formulation are rigorously assessed through independent validation cases against previously published results. The first validation case, shown in Figure 4, examines the predicted thermal conductivity as a function of graphene volume fraction and compares the present results directly with the experimental data reported in Ref. [41]. The material parameters adopted for this benchmark are specified as $\eta =0.43,\;L_{g}=1.7\;\mu m,\;t_{g}=60\;nm,\;K_{m}=0.2\;\frac{W}{m.K},\;\mathrm{and}\;R_{K}=3.5\times 10^{-9}\;\frac{m^{2}K}{W}$. The close correspondence between the present predictions and the published test results demonstrates that the proposed formulation can reliably reproduce the established thermal-conductivity trends over the investigated range of the graphene volume fraction.

**Figure 5:**
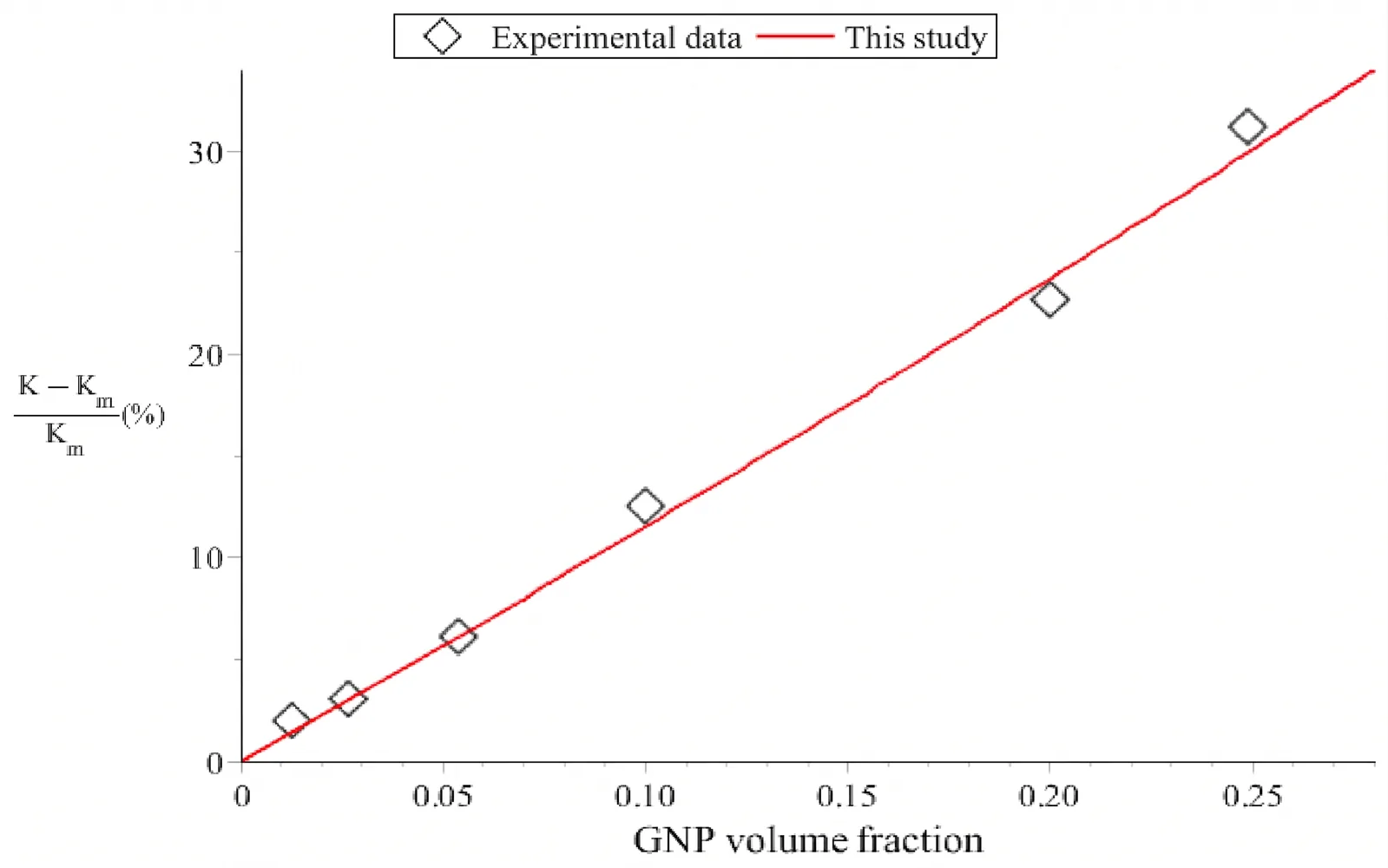
Validation of the proposed model against the experimental data for few-layer GNP-filled composites reported in Ref. [41].

To provide an independent assessment of the model’s predictive reliability, a second validation case is presented in Figure 5. In this comparison, the dependence of the composite thermal conductivity on graphene volume fraction is evaluated using a different set of material parameters, denoted as $L_{g}=0.35\;\mu m,\;t_{g}=1.7\;nm,$ $K_{m}=0.2\;\frac{W}{m.K},\;R_{K}=3.5\times 10^{-9}\;\frac{m^{2}K}{W},\;\mathrm{and}$ η = 0.83 and is subsequently benchmarked against the results in Ref. [41]. Despite the change in the adopted material parameters, the proposed formulation remains in close agreement with the reference results, confirming its consistency and robustness under different material conditions. As can be seen from Figure 4 and Figure 5, the micromechanical formulation accounts for the key factors governing thermal transport, including the intrinsic thermal conductivity, volume fraction and geometry of the nanofillers, as well as the interfacial thermal resistance. These parameters govern the heat-transfer pathways and effective thermal resistance of the composite, enabling the model to reproduce the published results based on physically meaningful mechanisms.

**Figure 6:**
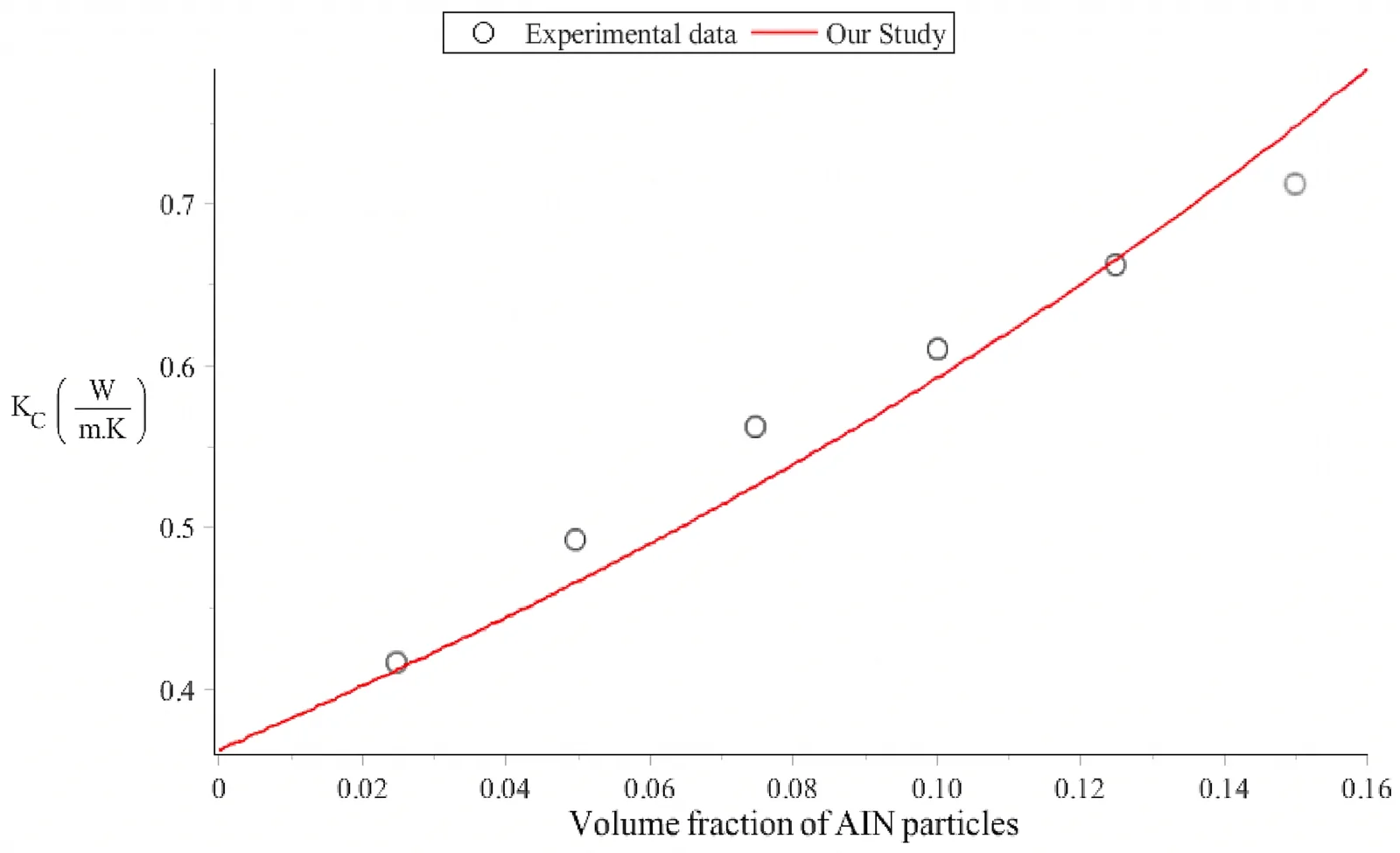
Thermal conductivity as a function of AlN volume fraction, compared with the experimental results in Ref. [36].

Further strengthening the validation of the proposed formulation, Figure 6 presents the predicted variation in the composite thermal conductivity with AlN volume fraction and provides a direct benchmark against the results reported in Ref. [36]. For simulation, it is considered that $hR_{1}$ = 5$k_{AlN}$. The predictions obtained from the present model exhibit excellent agreement with the published data throughout the investigated range of filler concentrations, closely reproducing both the magnitude and trend of the thermal conductivity.

### 3.2. Parametric Study

An extensive parametric study was conducted to systematically investigate the thermal transport behavior of a hybrid composite comprising a polymer matrix reinforced with GNPs and AlN particles. The primary objective was to quantify the influence of key material and geometrical parameters on the effective thermal conductivity of the hybrid composite system. Particular attention was given to the GNP volume fraction and geometrical characteristics including lateral dimensions and thickness, as well as the percentage and aspect ratio of the AlN particles. These parameters were varied systematically over the prescribed ranges to elucidate their individual contributions and the resulting changes in the overall thermal conductivity of the hybrid composite. In the micromechanical modeling procedure, GNPs are first dispersed within the polymer matrix, and the resulting effective matrix is subsequently reinforced by AlN microparticles. Thus, the individual contributions of both fillers to the effective thermal conductivity of composite materials are incorporated. Note that their direct interactions and the explicit formation of interconnected thermal-conduction networks are not directly simulated. To ensure a consistent basis for comparison and to isolate the effects of the investigated variables, the remaining material and model parameters were maintained at fixed values, as summarized in Table 1. It is noted that the present micromechanical method assumes a reasonably uniform filler dispersion and does not explicitly account for the processing-dependent effects. Thus, the micromechanical formulation is primarily intended to evaluate the influence of filler content, geometry, and interfacial thermal resistance under controlled dispersion conditions and to capture the general thermal-conductivity trends.

**Figure 7:**
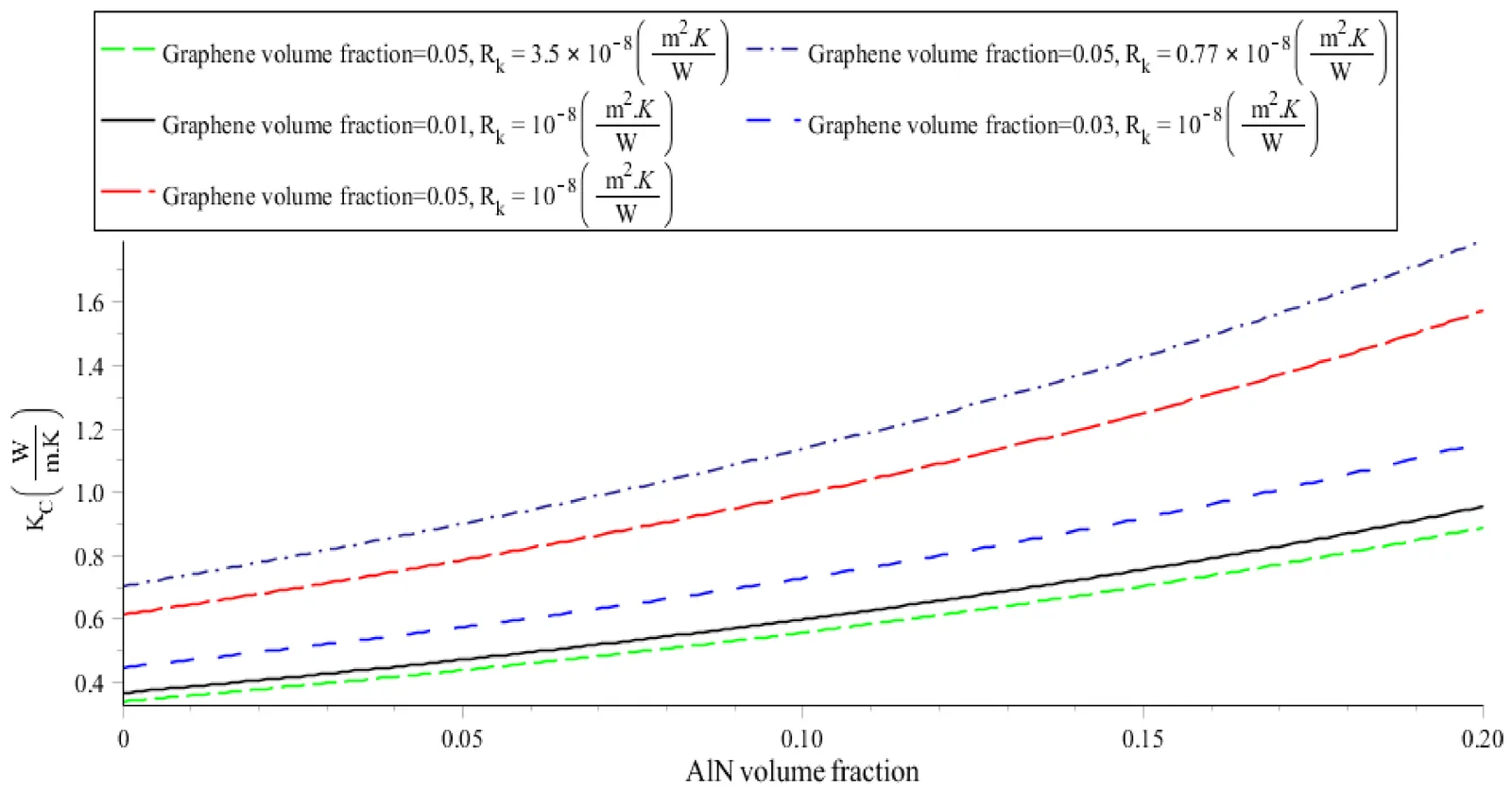
Effect of graphene volume fraction on the ternary composite thermal conductivity.

Figure 7 depicts the variation in the effective thermal conductivity of the hybrid composite with the volume fraction of AlN particles for different GNP contents. Also, the role of interfacial thermal resistance in the thermal properties is investigated. The results clearly demonstrate that increasing the AlN volume fraction leads to a progressive enhancement in the composite thermal conductivity. The increase in the effective thermal conductivity with increasing AlN content can be physically attributed to the much higher thermal conductivity of AlN compared with that of the matrix. When the microscale AlN percentage increases, a larger fraction of the low-conductivity matrix is replaced by highly conductive AlN microparticles, thereby providing additional heat-transfer pathways in the hybrid composite. Consequently, heat can be transferred more efficiently through the composite system. In addition, increasing the GNP volume fraction produces a further enhancement in thermal conductivity, highlighting the complementary contribution of the two thermally conductive fillers. The increase in the thermal conductivity with increasing graphene volume fraction is mainly attributed to the intrinsically high thermal conductivity of graphene and the formation of additional heat-transfer pathways within the polymer matrix. As the GNP content increases, the distance between neighboring GNPs decreases, facilitating heat transfer through the highly conductive graphene phase. Therefore, a higher GNP content results in more efficient heat transport and consequently, increases the effective thermal conductivity of the hybrid composite [11,27,42,43].

**Figure 8:**
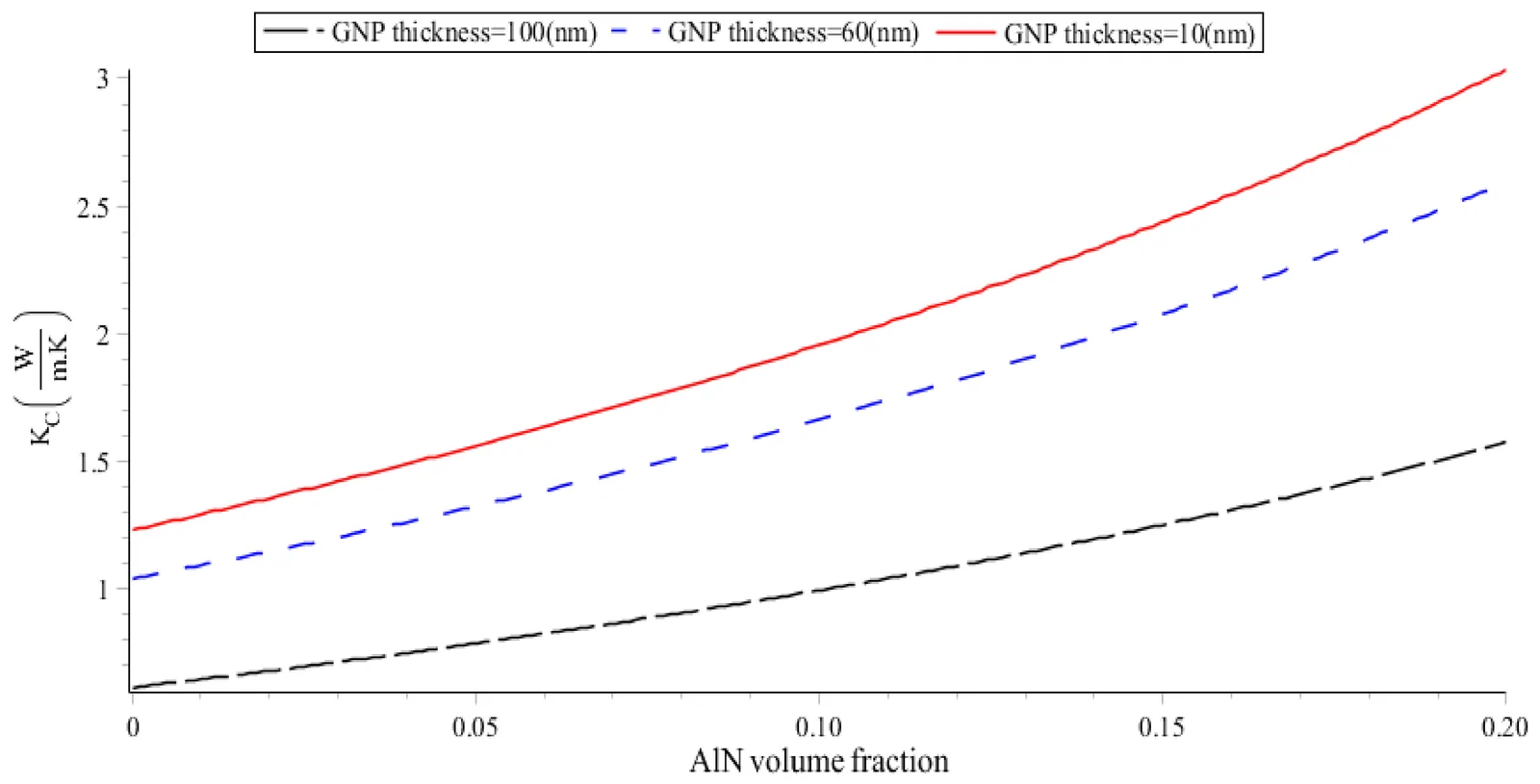
Effect of graphene thickness on the ternary composite thermal conductivity.

Figure 8 presents the dependence of the effective thermal conductivity of the GNP/AlN/polymer hybrid composite on the AlN particle volume fraction for different GNP thicknesses. A clear enhancement in thermal conductivity is observed with increasing AlN content, confirming the significant contribution of the highly conductive AlN particles to heat transport within the composite. The results show that increasing the GNP thickness results in a gradual reduction in the predicted thermal conductivity, whereas thinner GNPs provide a more pronounced improvement in thermal transport [19]. This behavior can be attributed to the more favorable heat-transfer pathways associated with thinner graphene fillers, which facilitate the transfer of thermal energy through the hybrid composite. It is clear from Equation (11) that the decrease in graphene thickness leads to an increase in the thermal conductivity of the GNPs, which finally improves the overall thermal conductivity of the hybrid composites.

**Figure 9:**
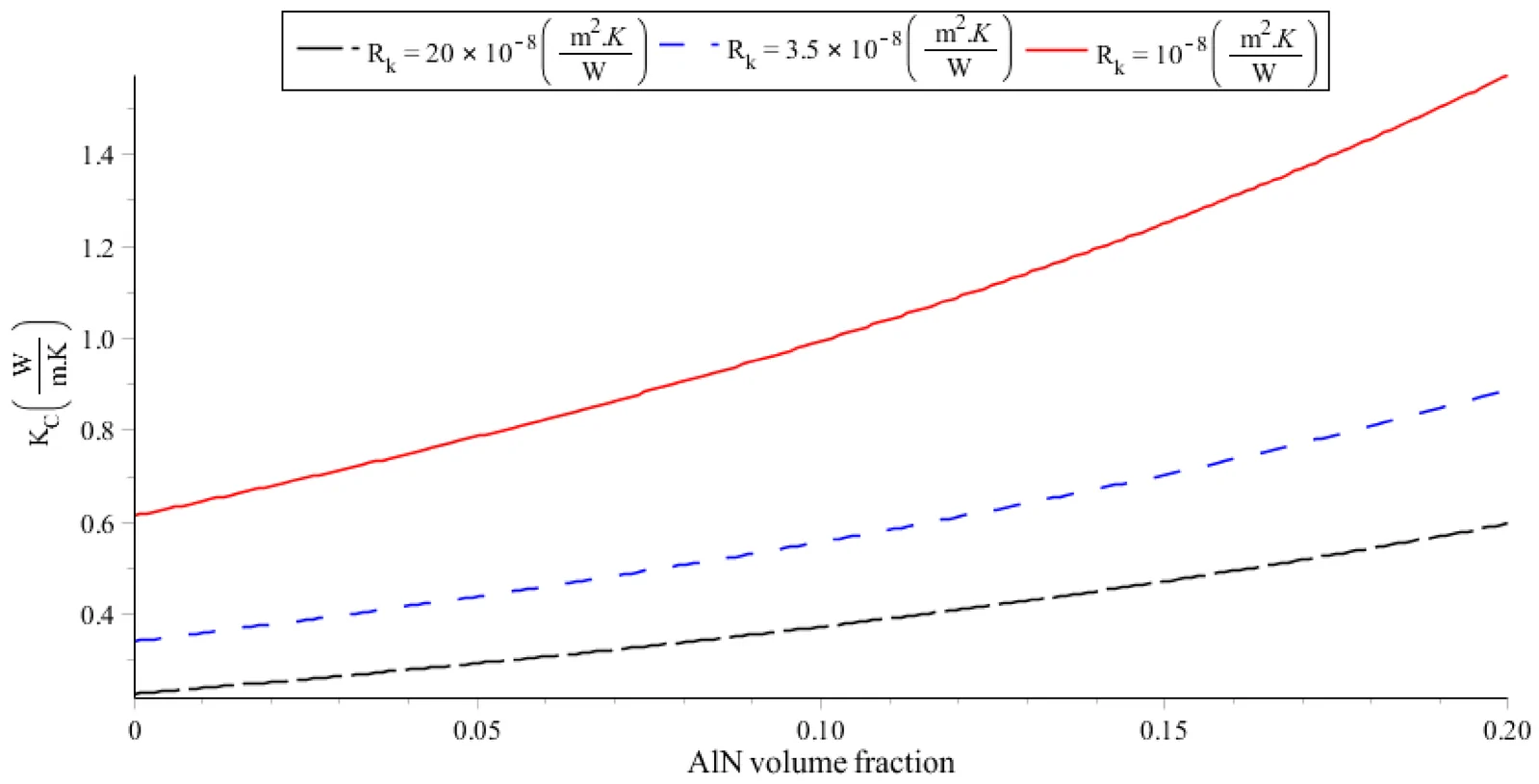
Effect of interfacial thermal resistance on the ternary composite thermal conductivity.

Figure 9 shows the variation in the effective thermal conductivity of the GNP/AlN/polymer hybrid composite with the AlN particle volume fraction for different values of interfacial thermal resistance, $R_{K}$. For a given AlN content, the thermal conductivity decreases systematically as $R_{K}$ increases. The observed reduction can primarily be attributed to the increased resistance to heat transfer across the GNP/polymer interface. As the interfacial thermal resistance increases, phonon transport across these interfaces becomes increasingly restricted, introducing additional thermal barriers within the composite [13,19,21,27].

**Figure 10:**
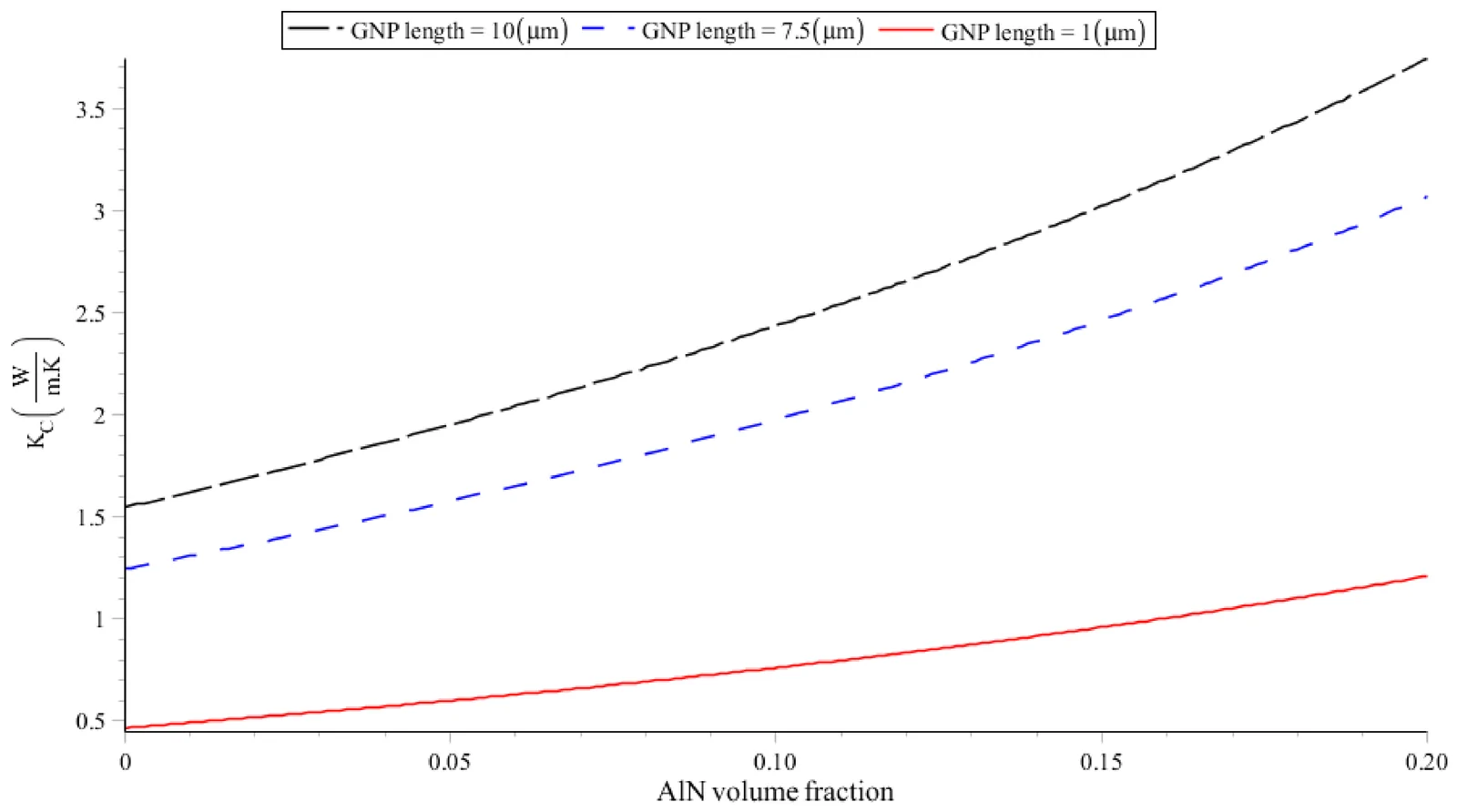
Effect of graphene lateral dimension on the ternary composite thermal conductivity.

**Figure 11:**
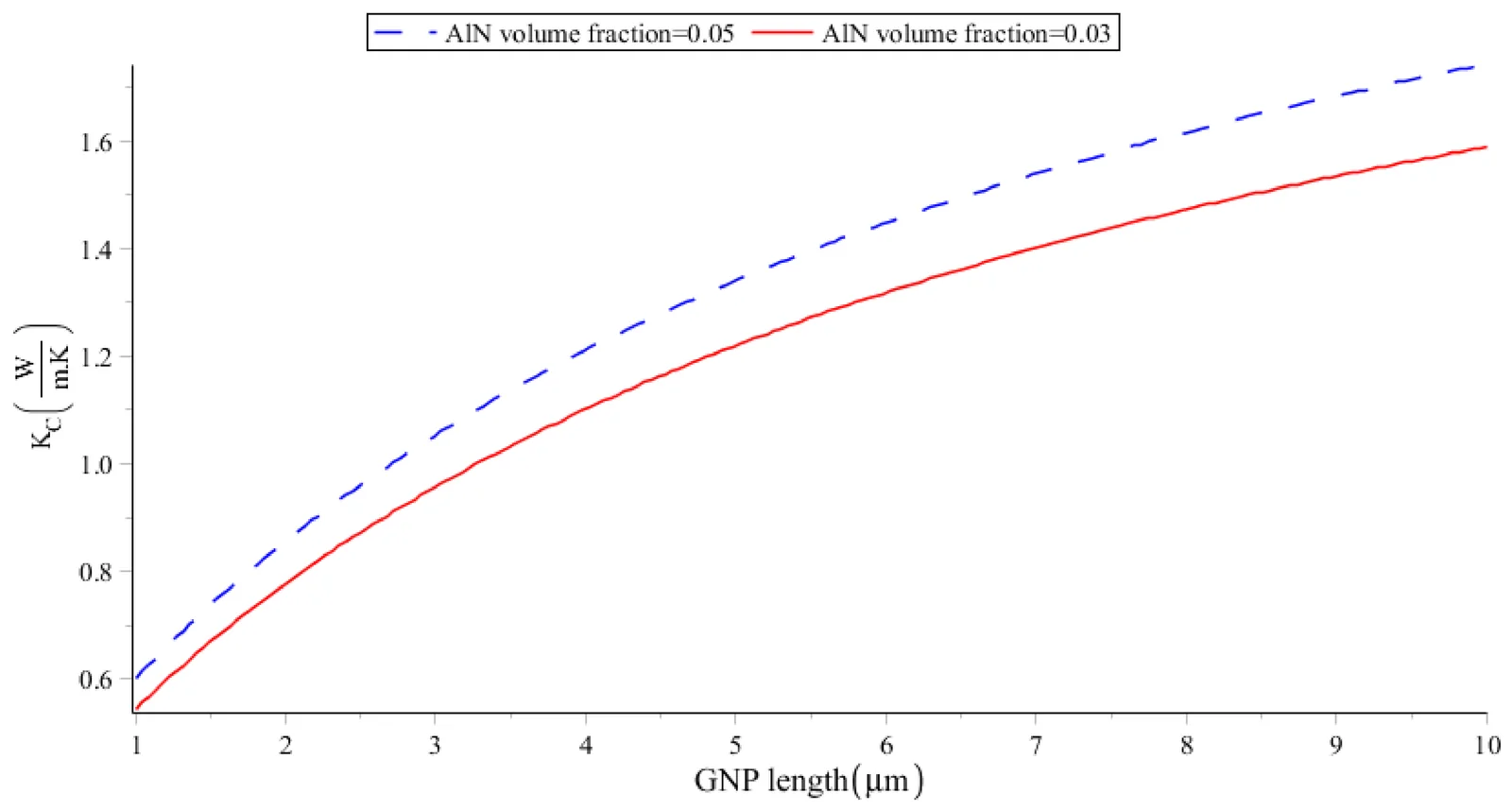
Variation in the ternary composite thermal conductivity with graphene lateral dimensions for different AlN volume fractions.

Figure 10 illustrates the effect of GNP lateral dimensions on the effective thermal conductivity of the GNP/AlN/polymer hybrid composite as a function of AlN volume fraction. The results demonstrate a pronounced enhancement in thermal conductivity with increasing GNP lateral size [19,21,22]. This behavior can be attributed to the ability of larger GNPs to establish more extensive and efficient heat-transfer pathways within the polymer matrix, thereby facilitating thermal energy transport across the composite. According to Equation (13), as the GNP length increases, the effective thermal conductivity of the polymer composite matrix is enhanced. Therefore, the lateral size of GNPs represents a key microstructural parameter for controlling the relative contributions of GNPs and AlN particles, and consequently, for tailoring the thermal conductivity of the hybrid composite. Figure 11 illustrates the variation in the thermal conductivity of the ternary composite as a function of graphene lateral dimensions for different AlN volume fractions.

**Figure 12:**
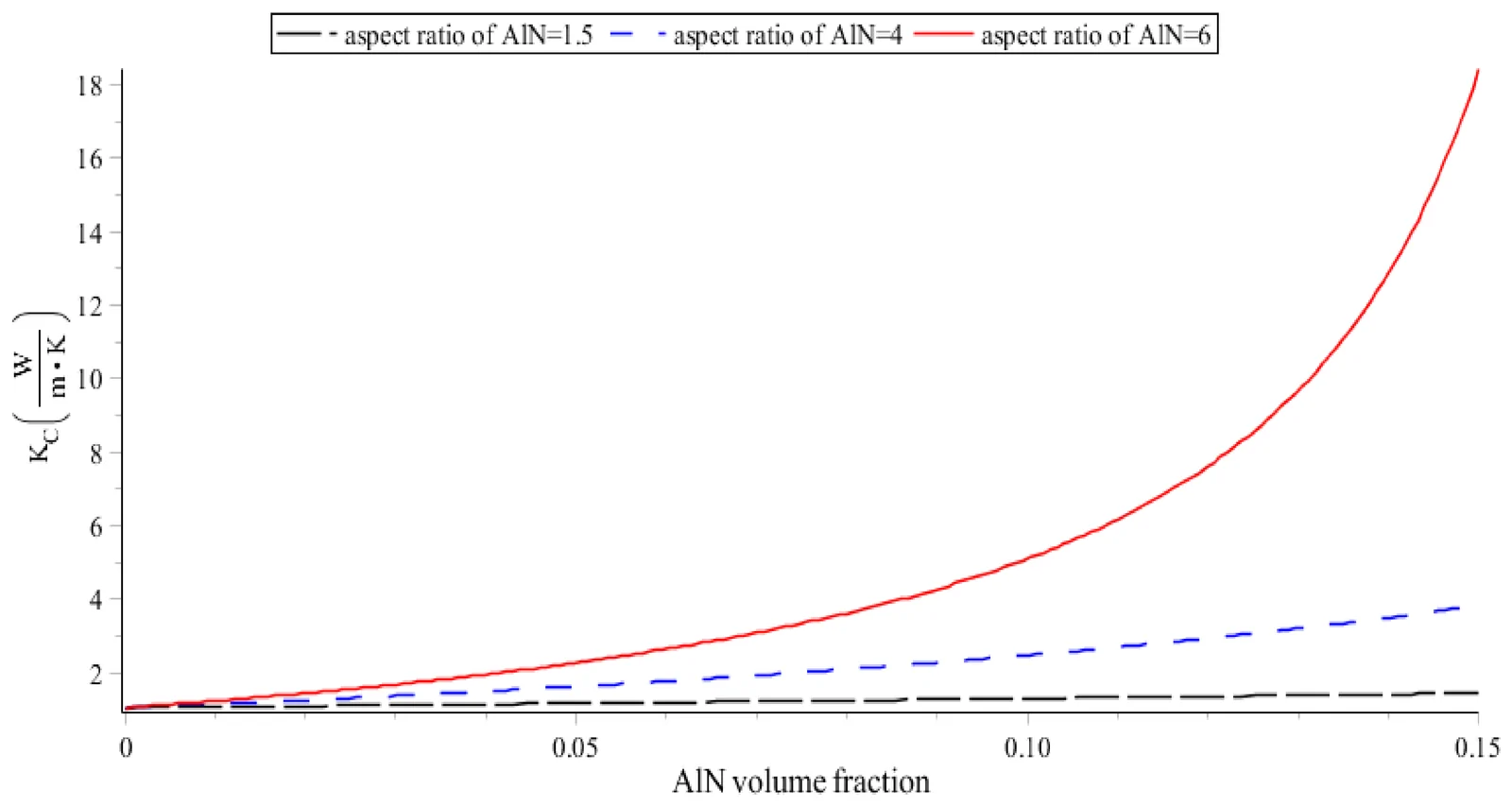
Effect of the AlN aspect ratio on the ternary composite thermal conductivity.

**Figure 13:**
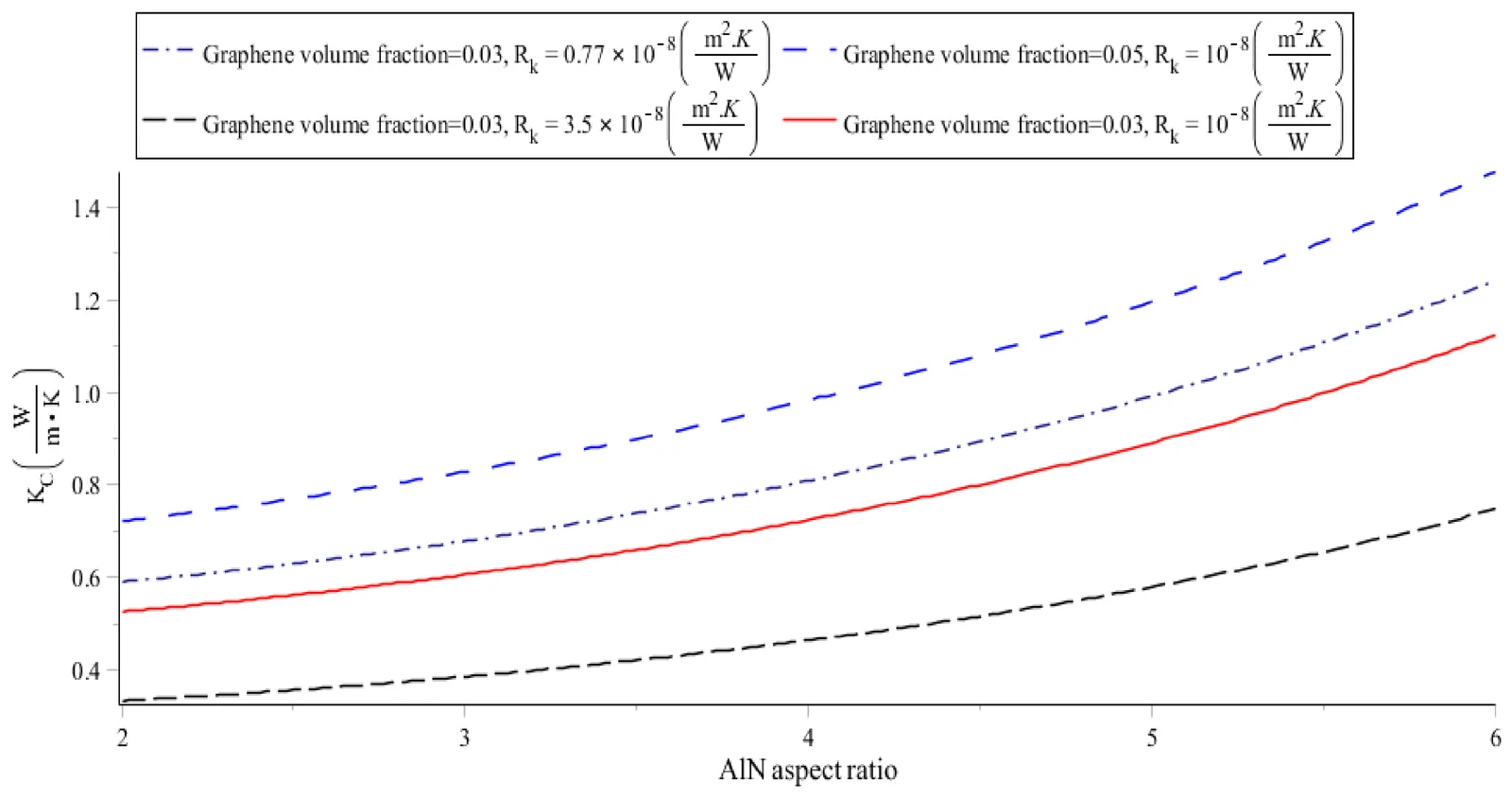
Variation in the ternary composite thermal conductivity with AlN aspect ratios for different graphene volume fractions.

Figure 12 examines the influence of the AlN particle aspect ratio on the effective thermal conductivity of the GNP/AlN/polymer hybrid composite. The results clearly indicate that increasing the AlN aspect ratio leads to a progressive enhancement in the composite thermal conductivity [42,43]. This behavior can be attributed to the elongated geometry of the highly conductive AlN particles, which promotes the formation of more efficient heat-transfer pathways within the matrix. Figure 13 presents the variation in the thermal conductivity of the ternary composite with the AlN aspect ratio for various graphene volume fractions.

The GNP/AlN/polymer hybrid composites may be promising for lightweight thermal-management applications requiring enhanced thermal conductivity. Potential applications include electronic packaging, power-electronics components, thermal interface materials, and lightweight heat-spreading structures [44,45,46,47,48,49,50,51]. The hierarchical combination of these multiscale fillers may improve thermal transport without requiring excessive GNP loading or AlN content. Thus, the proposed hybrid composite architecture may provide a tunable approach for developing polymer-based materials for advanced thermal-management applications.

## 4. Conclusions

In this study, a comprehensive micromechanical investigation was performed to evaluate the effective thermal conductivity of a ternary GNP/AlN/polymer composite. A hierarchical micromechanical formulation was developed to characterize the thermal transport behavior of the hybrid composite system and to clarify the effects of the principal material and geometrical parameters. Based on the obtained results, the following conclusions can be drawn:

• Effect of GNP content: Owing to their exceptionally high intrinsic thermal conductivity, GNPs significantly enhance heat transport within the polymer matrix by providing efficient conductive pathways. Increasing the GNP volume fraction results in a progressive increase in the effective thermal conductivity of the ternary composite.
• Effect of GNP thickness: The effective thermal conductivity exhibits a strong dependence on GNP thickness. An increase in GNP thickness leads to a reduction in the composite thermal conductivity, whereas thinner GNPs provide a more pronounced enhancement. This behavior indicates that optimizing the thickness of GNPs is essential for establishing efficient heat-transfer pathways within the polymer matrix.
• Effect of GNP lateral dimension: Larger GNP lateral dimensions lead to higher thermal conductivity of graphene/AlN/polymer composites by promoting the formation of more extended and efficient heat-transfer pathways.
• Effect of AlN content: Increasing the AlN volume fraction further improves the thermal conductivity of the hybrid composite due to the high thermal conductivity of AlN particles.
• Effect of AlN aspect ratio: Increasing the aspect ratio of AlN fillers produces a noticeable enhancement in the thermal conductivity. The presence of high-aspect-ratio AlN particles and GNPs generates efficient heat-conduction pathways, resulting in enhanced thermal performance compared with systems containing either filler individually.
• Effect of interfacial thermal resistance: Increasing the interfacial thermal resistance reduces the effective thermal conductivity of the graphene/AlN/polymer composite by restricting heat transfer. Minimizing interfacial thermal resistance is therefore crucial for exploiting the full thermal-conductivity potential of the hybrid filler system.

## Figures and Tables

**Table 1:** Parameters used in the present study for the predictions of thermal conductivity of the ternary composites.

λ	$K_{m}$ $\left(W/m.K\right)$	$K_{AlN}$ $\left(W/m.K\right)$	η	$R_{K}$ $\left(m^{2}.K/W\right)$	$t_{g}$ $\left(nm\right)$	$L_{g}$ $\left(\mu m\right)$
2.65	0.2	160	0.43	$10^{-8}$	60	1.7

## Data Availability

Data will be made available on request.
